# Survival Analysis of ACL Graft and Contralateral ACL Ruptures in Patients Younger Than 18 Years

**DOI:** 10.1177/23259671251317490

**Published:** 2025-03-03

**Authors:** Kate E. Webster, Julian A. Feller

**Affiliations:** † School of Allied Health, Human Services and Sport, La Trobe University, Melbourne, Victoria, Australia; ‡ OrthoSport Victoria, Epworth HealthCare, Melbourne, Australia; Investigation performed at OrthoSport Victoria and La Trobe University, Melbourne, Australia

**Keywords:** anterior cruciate ligament, reconstruction, athlete, sports injury

## Abstract

**Background:**

Although high rates of graft and contralateral anterior cruciate ligament (ACL) ruptures have been reported in younger patients after ACL reconstruction (ACLR), recent evidence suggests that previously reported crude event rates underestimate the actual event risk.

**Purpose:**

To report rates of graft and contralateral ACL rupture after ACLR in a large series of younger patients using survival analysis.

**Study Design:**

Cohort study; Level of evidence, 3.

**Methods:**

Patients aged <18 years at the time of primary ACLR were identified from a single-surgeon database over 12 years ending January 2018. Patients with a previous contralateral ACL rupture or bilateral ACL ruptures were excluded. Overall, 388 patients (204 males, 184 females) were included in the final dataset. Bespoke survey data and clinic follow-up data were used to record graft rupture and contralateral ACL rupture events. Rates of graft and contralateral ACL rupture were calculated using Kaplan-Meier survival analysis. Log-rank tests were used to compare survival functions between several subgroups.

**Results:**

According to Kaplan-Meier survival analysis, the cumulative rates at 2, 5, and 10 years for graft rupture were 11%, 17%, and 22%, and the cumulative rates for contralateral ACL injury were 7%, 19%, and 33%. Males had significantly greater rates of graft rupture than females throughout a 10-year follow-up period (*P* < .001). Contralateral ACL rupture survival functions were not significantly different between the sexes, although rates were higher in females until 5 years postoperatively, after which contralateral ACL ruptures increased in males, with a cumulative rate of 39% at 10 years compared with 29% for females. Survival rates did not vary between different age groups (<16 vs ≥16 years), but females with a graft diameter of <7 mm on the femoral side had significantly greater graft rupture rates than females with grafts ≥7 mm (*P* = .04).

**Conclusion:**

The present study is one of the largest consecutive series of younger patients, with one of the longest follow-up periods, reporting a high risk for a second ACL injury. Over time, the cumulative risk for contralateral ACL rupture was higher than for graft rupture. In males, the risk for contralateral ACL rupture continued to increase after 5 years.

Younger age is a recognized risk factor for sustaining multiple anterior cruciate ligament (ACL) injuries.^10,14,18,24,29,32,38^ This has been consistently determined via large-scale registry datasets^14,18^ as well as institutional cohort studies^23,26,33^ and may include rupture of an ACL graft or a subsequent contralateral ACL injury after a first-time ACL reconstruction (ACLR). The risk for either of these events occurring has been estimated to be between 2 to 5 times greater in younger patients compared with their older counterparts.^14^ While the definition of younger has varied, the proportion of ACL-injured patients who are <25 years old who sustain multiple ACL injuries has been documented^11,26,28,33^ at between 20% to 50%. Such high rates of multiple ACL injuries in younger patients are of significant concern. Even more concerning is that many of these studies report crude event proportions at a mean or median follow-up time, which has been shown to underestimate the actual event risk.^6^

The significant and negative effect of age is also clearly apparent when patients are followed over a longer period after ACLR. In a longitudinal study by Salmon et al,^27^ graft survival was 85% for adult patients compared with only 61% for adolescent athletes (aged ≤18 years) at a 20-year follow-up. This was despite both groups having similar rates of returning to sports and current levels of activity. Despite the limitation of small group numbers (39 adolescents in total; 13 males and 26 females), graft survival was reported at only 46% at 20 years for the adolescent males, compared with 69% for adolescent females. This sex-based disparity, which suggests that males are at higher risk for graft rupture than females, has also been shown in other cohort studies.^5^ Other research has indicated that females have significantly higher rates of subsequent contralateral ACL injuries.^24,31^

A recent large study in pediatric patients by DeFrancesco et al^7^ showed significantly higher rates of multiple ACL injuries with the use of survival analysis than had previously been reported. In a sample of 504 patients <16 years, the cumulative rate of graft rupture was 21.6% by 4 years, which was notably higher than the rate of 8.7%, which had previously been reported in a meta-analysis of pediatric patients with a mean age of 13 years (range, 9-16 years).^39^ Collectively, the results of both Salmon et al^27^ and DeFrancesco et al^7^ highlight the importance of the type of analysis performed as well as the assessment of younger patients over a longer time frame. Unfortunately, there is a lack of other similar studies.

The present study was undertaken to report rates of graft and contralateral ACL rupture after ACLR in a large series of younger patients (<18 years) using survival analysis up to 10 years after primary ACLR.

## Methods

### Patient Selection

A consecutive cohort of 400 patients who had undergone primary autograft ACLR between December 2004 and February 2018 by a single experienced knee surgeon (J.A.F.). The inclusion criteria were patients who were <18 years at the time of surgery. Patients with a previous contralateral ACL rupture (n = 8) or bilateral ACL ruptures (n = 2) were excluded, as were 2 patients who died within 6 months of surgery, leaving 388 eligible patients (203 males, 185 females) in the final study cohort.

After receiving ethics committee approval, we contacted all patients for inclusion in the present study. Some patients had been included in previous studies related to risk factors for second ACL injury^32^ or exploring ACL injuries in younger patients.^33^ Others were also part of a longitudinal study investigating comprehensive outcomes after ACLR.^34,35^

### Surgical and Rehabilitation Details

All ACLRs were performed using a variety of graft types, but predominately hamstring tendon autografts. No allografts were used. Regardless of graft type, fixed-loop suspensory fixation was used on the femoral side and interference screw fixation on the tibial side, except in patients with clearly open growth plates. In the latter group, tibial fixation was performed using nonabsorbable sutures tied to a fixation post. During the surgical period, lateral extra-articular tenodesis was not routinely used (only 5 patients had anterolateral augmentation, and this was performed using a modified Ellison procedure^1,8^).

Patients were discharged from the hospital on the first postoperative day, with weightbearing allowed as tolerated. Braces were not routinely used, the exceptions being when extensive meniscal tears were repaired. In this situation, a knee immobilizer was used for 3 weeks, during which time the patient remained partially weightbearing. Patients completed rehabilitation at a clinic of their choice, and standard rehabilitation protocols and guidelines were provided that encouraged immediate full active knee extension and the restoration of quadriceps function as soon as possible.^3^ Progression depended on recovery of range of motion, strength, and the absence of an effusion. In general, running did not commence until 3 to 4 months postoperatively with sports-specific activities commencing 1 to 2 months later. Clearance to return to competitive sports was typically between 9 and 12 months after surgery and was determined by the treating surgeon. The patient had to complete 1 month of completely unrestricted full-contact training before resuming competition.

### Data Collection

Prospectively collected patient databases were searched for demographic, injury, and surgical information. Demographic characteristics included age at surgery, sex, and a previous history of contralateral ACL injury. The primary variables of interest for this study were the number of second ACL injuries, either ACL graft rupture or contralateral ACL injury after the primary procedure. Medical records of the entire cohort were initially checked beginning February 2023 to identify patients who had sustained a second ACL injury and the number and type (graft rupture, contralateral ACL) of injury were recorded. Patients who were identified from their medical record as having had both a graft rupture and contralateral ACL injury were not contacted further, as the purpose of this study was to document subsequent injuries rather than the outcome of the treatment of those injuries. The remaining patients were individually contacted via telephone or email/electronic survey to identify any further ACL injuries in the study cohort. Both forms of contact required the patient (or parent) to answer structured questions regarding any further injuries to the ACL-reconstructed knee or the contralateral knee.^32^ The questions included the date of further injury (separate questions for graft rupture and contralateral ACL injury), activity at the time of injury, and if sports-related, which sport and whether the sport was a contact or noncontact sport. Finally, the patient indicated the details of any medical treatment sought. Each patient’s last follow-up date was considered to be the day of their last clinic review/visit or the day they completed the survey/telephone interview, whichever was the latest. By the time of the follow-up, at least 5 years had elapsed since their index ACLR in all patients.

### Statistical Analysis

Rates of ACL graft rupture and contralateral ACL injuries were calculated using Kaplan-Meier survival analysis, with patients censored after their last known follow-up. Log-rank tests were used to compare survival functions between several subgroups (sex, age, and graft diameter). Statistical significance was set at *P* ≤ .05, and all analyses were performed using SPSS software Version 28 (IBM Corp).

## Results

**Figure 1.**
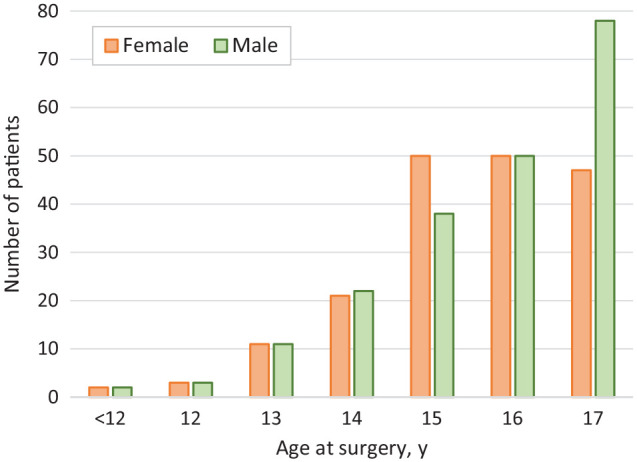
Histogram of patient age at surgery. Open growth plates were present in 54 patients (14%) and closed/closing growth plates in 334 patients (86%).

The mean follow-up period was 8 years (range, 0-18 years). One patient had not attended any postoperative appointments and did not respond to any follow-up attempt, and 17 patients did not have follow-up data beyond 12 months postoperatively. One patient had an early graft rupture at 6 months and underwent revision surgery but did not attend any follow-up appointments beyond 3 months after revision. For 50% of the cohort, the follow-up was between 6 and 10 years. A minimum 3-year follow-up was achieved for 91% of the cohort and a minimum 5-year follow-up was achieved for 83% of the cohort. Descriptive data for the cohort is shown in Table 1, and a histogram of patient age is shown in Figure 1.

**Table 1:** Patient Characteristics Overall and According to Sex^*a*^

	Overall	Female	Male
	(N = 388)	(n = 184)	(n = 204)
Age, y	16.1 (9.9-17.9)	16.0 (9.9-17.9)	16.3 (11.7-17.9)^*b*^
Graft type			
Hamstring	352	174	178
Patellar tendon	16	3	13
Quadriceps	20	7	13
Graft size diameter, mm			
Femoral side	7.8 (6-11)	7.4 (6-9.5)	8.1 (6-11)^*c*^
Tibial side	8.4 (6.5-12)	8.1 (6.5-10)	8.8 (6.5-12)^*c*^
Medial meniscal tear	86	51	35^*b*^
No treatment	18	9	9
Repair	43	27	16
Resect	25	15	10
Lateral meniscal tear	133	59	74
No treatment	63	27	36
Repair	13	9	4
Resect	57	23	32^*d*^

a Values are presented as mean (range) or number of patients. Significant differences between male and female patients: ^*b*^*P* < .05, ^*c*^*P* < .001.

d Two resections during a previous surgery.

**Figure 2.**
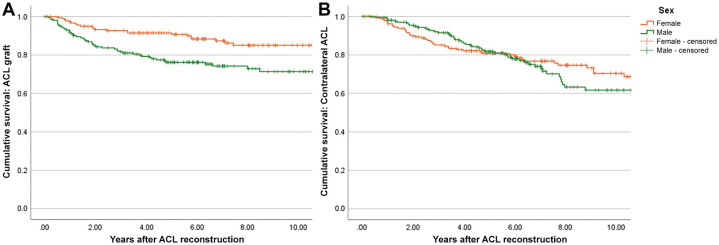
Kaplan-Meier survival curves of (A) graft rupture and (B) contralateral ACL rupture according to sex in patients having undergone ACLR. ACL, anterior cruciate ligament; ACLR, anterior cruciate ligament reconstruction.

**Figure 3.**
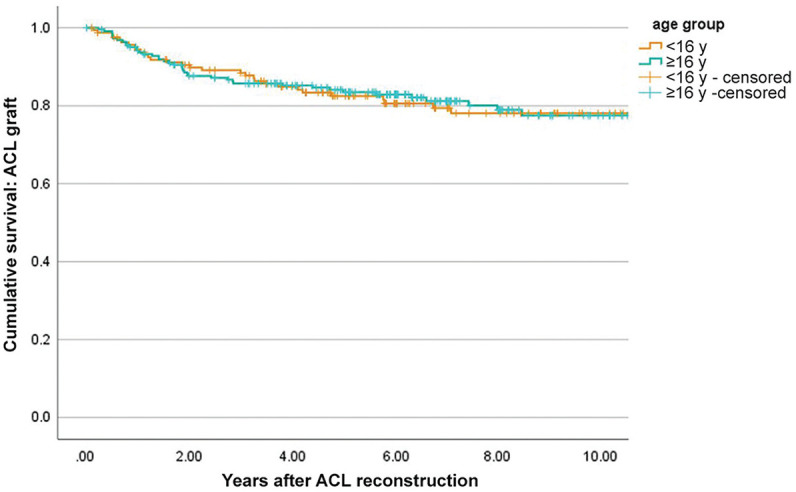
Kaplan-Meier survival curves of graft rupture according to age grouping.

Based on Kaplan-Meier survival analysis, the cumulative rates of graft rupture via survival analysis were 11% at 2 years, 17% at 5 years, and 22% at 10 years (Table 2). This varied by sex, in which males had significantly greater rates of graft rupture than females (log-rank, *P* = .001) throughout a 10-year follow-up period (Figure 2A). For contralateral ACL injury, the cumulative rates were 7% at 2 years, 19% at 5 years, and 33% at 10 years. Contralateral ACL rupture survival functions were not significantly different between male and female patients (log-rank, *P* = .7), although rates were higher in females until 5 years, after which males continued to sustain contralateral ACL ruptures, with a cumulate rate of 39% at 10 years compared with 29% for females (Figure 2B). There were no differences in survival rates between patients who were <16 versus ≥16 years for graft rupture (log-rank, *P* = .92) (Figure 3) or contralateral ACL injury (log-rank, *P* = .94).

**Table 2:** Rates of ACL Graft Rupture and Contralateral ACL Rupture at 1-, 2-, 5-, and 10-Year Follow-up^*a*^

Years After ACLR	Graft Rupture			Contralateral ACL Rupture		
	Overall	Female	Male	Overall	Female	Male
1	5.8 (3.8-8.6)	2.8 (1.2-6.5)	8.5 (5.4-13.3)	2.4 (1.2-4.5)	4 (1.9-8)	2 (0.8-5.3)
2	11.2 (8.4-14.8)	6.7 (3.9-11.5)	15.3 (11-21.2)	7.3 (5.5-10.4)	10.1 (6.5-15.5)	4.7 (2.5-8.8)
5	16.9 (13.4-21.2)	9.2 (5.7-14.6)	23.9 (18.4-30.8)	18.7 (15-23.3)	19.2 (14-25.9)	18.4 (13.4-25.1)
10	22.1 (17.7-27.5)	14.8 (9.8-22)	28.8 (22.1-37.1)	33.3 (27.6-39.9)	29.4 (22.2-38.3)	38.7 (29.9-49)

a Values are presented as percentages (95% CI). ACL, anterior cruciate ligament; ACLR, anterior cruciate ligament reconstruction.

**Figure 4.**
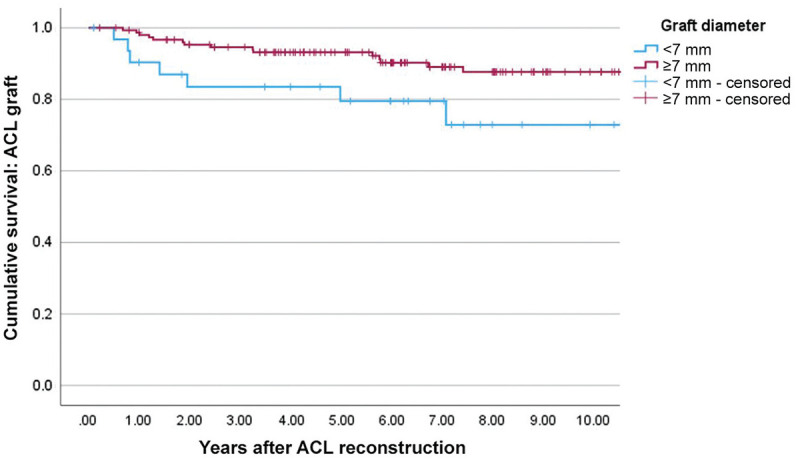
Kaplan-Meier survival curves of graft rupture in female patients according to graft diameter on the femoral side.

For the entire patient cohort and male patients, graft survival rates were not significantly different between patients who had smaller (<7 mm) compared with larger (≥7 mm) sized grafts (log-rank, *P* > .05). However, female patients with a smaller-sized graft on the femoral side had significantly greater rates of graft rupture than those with a larger-sized graft (log-rank, *P* = .04) (Figure 4).

## Discussion

The present study's findings confirm the high risk of a second ACL injury in younger patients. Over time, we found that the cumulative risk for contralateral ACL rupture was higher than that for graft rupture. This contradicts some previous work in adolescent cohorts. Salmon et al^27^ and DeFrancesco et al^7^ showed greater graft rupture rates compared with contralateral ACL injury in their young patient cohorts. In the present study, females initially had higher rates of contralateral ACL rupture, but at around 5 years postoperatively the survival curves crossed over, and after 5 years, males had higher rates of contralateral ACL injuries. This was not seen in Salmon et al^27^ despite their having a 20-year follow-up period, although it was most likely due to their small sample size of 13 adolescent male patients. An earlier 15-year follow-up study by the same research group did, however, show that male patients had increased rates of contralateral ACL injury after 5 years compared with females,^19^ consistent with the results of the present study. Morgan et al^19^ suggested that more young male patients may have returned to sports that have a higher risk for a knee injury and that this likely contributed to their finding of increased contralateral ACL injuries in this cohort. Interestingly, in their study, no contralateral ACL injuries occurred in the female participants after 6 years whereas in the present study, females continued to experience contralateral ACL injury between 5 and 10 years, just at a slower rate than their male counterparts. DeFrancesco et al^7^ had only a 4-year follow-up period and as such did not assess their young patients for a long enough period for this trend in contralateral ACL injuries to emerge.

The most incremental cumulative increase in graft ruptures was seen within the first 3 years, particularly for male athletes (shown in Figure 1). This is consistent with data from registry studies, which show that the incidence of ACL revision surgery peaks at 1 to 2 years^14^ and raises several issues, particularly in terms of when young athletes should return to sports. Return-to-sport criteria have been extensively discussed, but overall, there is little evidence that criteria can be directly applied to young athletes to prevent further injury.^15,37^ While the recovery of quadriceps strength is important during ACL rehabilitation and may allow for an athlete to successfully return to sports, recent studies have either not found a relationship between the recovery of strength and risk for subsequent ACL injury in young athletes or have found that athletes with higher strength may have a higher injury risk.^4,30^ It has also been suggested that it may be beneficial for young athletes to delay their return to strenuous sports.^21^ However, a recent study in which young athletes (<20 years at surgery) were advised to delay their return to competitive sports until 12 months after a first-time ACLR found that there was no beneficial effect of this delay. At 3 to 5 years after reconstruction, 33% of patients in the delayed group had suffered a subsequent ACL injury compared with 31% in a group who were not advised to delay their return to sports.^36^

Most patients in the present study received hamstring tendon grafts. Results from several large data sets have shown higher revision rates for hamstring tendons compared with patellar tendon grafts.^13,25^ Data from the Norwegian ACL registry showed an increased risk of revision after the use of hamstring grafts compared with patellar tendon grafts, particularly for the youngest patients (15-19 years).^25^ Data from the Kaiser Permanente Registry similarly showed a significantly higher risk of rupture (hazard ratio, 1.6) for hamstring grafts compared with patellar tendon grafts in patients <21 years at surgery.^17^ In the recent STABILITY trial, the addition of a lateral extra-articular tenodesis (LET) to a single-bundle hamstring graft in patients ≤25 years was shown to significantly reduce the rate of graft rupture from 11% in the group without a LET to 4% in the LET group.^9^ Unfortunately, this variable could not be evaluated because a LET procedure was not routinely used in the present cohort. In the present study, females with a smaller graft diameter on the femoral side had greater rates of graft rupture. Previous studies have similarly shown statistical associations between a small graft diameter at surgery and subsequent graft rupture.^12,16^ However, collectively, the literature is mixed, and this association has not been consistently reported. A recent systematic review evaluated the risk of ACLR failure between various dichotomous graft size groupings and found that of 8 comparisons, graft diameters of <7 mm were associated with significantly higher failure rates.^2^ This review included patients of all ages and activity levels. Data from the New Zealand ACL Registry found no relationship between graft diameter and graft failure in patients <20 years at the time of surgery.^20^ In the present study it is unclear why an association between graft diameter and graft rupture was seen for only the female patients and not the young males. Overall, further data are required to understand the role of graft diameter in graft failure in young active patients.

Survival rates of graft or contralateral ACL rupture were no different between relatively younger (<16 years) and older (≥16 years) adolescent patients in the present study. Patel et al^22^ found that patients <15 years had 3.1 times higher odds of contralateral ACL rupture than those aged 15 to 20 years at the time of surgery. The mean follow-up time was, however, only 4 years, and as such it is unknown whether such a difference would persist over time. The present findings, in which the mean follow-up time was 8 years, indicate that the risk for multiple ACL injuries continues to remain present and meaningfully accumulates over a significant period for young athletes. This may have negative consequences for the ability of a young athlete to continue to participate in sports and physical activity over the lifespan as well as for the long-term health of the knee joint.

### Strengths and Limitations

As with all research, the present study has both strengths and limitations. Strengths include a consecutive cohort of young athletes all operated on by a single experienced knee surgeon, as well as the long follow-up period. Limitations include the pragmatic nature of the study; as such, we did not routinely record the sports participation levels of patients over time. We therefore do not explicitly know the timing of return to play, how long patients continued to play after surgery, or if patients changed from a high-risk sport for knee injuries to a lower one. These are all factors that have an impact on exposure and thus the risk for reinjury. The high reinjury rates that we saw over time would nonetheless indicate that these young patients did remain highly active after their primary ACLR.

## Conclusion

The present study is one of the largest consecutive series of younger patients, with one of the longest follow-up periods, reporting a high risk for a second ACL injury. Over time, the cumulative risk for contralateral ACL rupture was higher than for graft rupture. Understanding the postoperative time course for second ACL injury in young patients is important so that factors that contribute to this increased risk can be better understood and such injuries prevented.
